# Human endometrial mesenchymal stem cells restore ovarian function through improving the renewal of germline stem cells in a mouse model of premature ovarian failure

**DOI:** 10.1186/s12967-015-0516-y

**Published:** 2015-05-12

**Authors:** Dongmei Lai, Fangyuan Wang, Xiaofen Yao, Qiuwan Zhang, Xiaoxing Wu, Charlie Xiang

**Affiliations:** The International Peace Maternity and Child Health Hospital, School of Medicine, Shanghai Jiaotong University, Shanghai, 200030 China; State Key Laboratory for Diagnosis and Treatment of Infectious Diseases, the First Affiliated Hospital, Zhejiang University School of Medicine, Hangzhou, 310003 China

**Keywords:** Endometrial mesenchymal stem cells (EnSCs), Menstrual blood, Ovarian function, Premature ovarian failure/insufficiency (POF/POI), Chemotherapy, Germline stem cells (GSCs)

## Abstract

**Background:**

Human endometrial mesenchymal stem cells (EnSCs) derived from menstrual blood have mesenchymal stem/stromal cells (MSCs) characteristics and can differentiate into cell types that arise from all three germ layers. We hypothesized that EnSCs may offer promise for restoration of ovarian dysfunction associated with premature ovarian failure/insufficiency (POF/POI).

**Methods:**

Mouse ovaries were injured with busulfan and cyclophosphamide (B/C) to create a damaged ovary mouse model. Transplanted EnSCs were injected into the tail vein of sterilized mice (Chemoablated with EnSCs group; n = 80), or culture medium was injected into the sterilized mice via the tail vein as chemoablated group (n = 80). Non-sterilized mice were untreated controls (n = 80). Overall ovarian function was measured using vaginal smears, live imaging, mating trials and immunohistochemical techniques.

**Results:**

EnSCs transplantation increased body weight and improved estrous cyclicity as well as restored fertility in sterilized mice. Migration and localization of GFP-labeled EnSCs as measured by live imaging and immunofluorescent methods indicated that GFP-labeled cells were undetectable 48 h after cell transplantation, but were later detected in and localized to the ovarian stroma. 5’-bromodeoxyuridine (BrdU) and mouse vasa homologue (MVH) protein double-positive cells were immunohistochemically detected in mouse ovaries, and EnSC transplantation reduced depletion of the germline stem cell (GSCs) pool induced by chemotherapy.

**Conclusion:**

EnSCs derived from menstrual blood, as autologous stem cells, may restore damaged ovarian function and offer a suitable clinical strategy for regenerative medicine.

**Electronic supplementary material:**

The online version of this article (doi:10.1186/s12967-015-0516-y) contains supplementary material, which is available to authorized users.

## Background

Cancer patients—especially women younger than 40 years of age—who receive chemotherapy or radiation often suffer reproductive damage. This damage is frequently associated with premature ovarian failure/insufficiency (POF/POI) and infertility due to ovarian germ cell toxicity. Alkylating agents such as cyclophosphamide (Cy) carry the greatest risk of POF/POI of all chemotherapeutic drug classes [1-3]. Recently Kalich-Philosoph and colleagues reported that Cy treatment activates growth of the quiescent primordial follicle population in mice and depletes the ovarian reserve, leading to early ovarian failure and infertility [4]. Busulfan, another antineoplastic alkylating agent, is known to affect female reproduction via ovarian cytotoxicity. A single injection of 40 mg/kg busulfan in mice leads to small follicle depletion and completely oocyte loss and it is dose-dependent [5, 6]. In rats, primordial and primary ovarian follicles of offspring were affected and caused oocyte loss and infertility by maternal treatment with busulfan [7]. It is primarily believed that dormant primordial follicles were a nonrenewable population representing the “ovarian reserve” of reproductive potential [8]. Activation of the primordial follicle initiates unidirectional and irreversible growth, inducing either ovulation or atresia [9]. Thus, long-term maintenance of most follicles in a dormant state is important to preserve the primordial follicle stockpile and restore ovarian function during cancer treatment.

Stem cells are attractive because they are self-renewing and have the potential to differentiate into all three germ layers. Recent interest in the therapeutic potential of stem cells has grown and multipotent stem cells could be developed from different sources, such as bone marrow [10-12], adipose tissue [13, 14], amniotic fluid [15], and the amnion [16], and all have been shown to have the potential to restore ovarian function and rescue long-term fertility in chemotherapy-treated female mice.

Recently, a novel stem cell source has the superiority to conventional sources. Human endometrial mesenchymal stem cells (EnSCs) with mesenchymal stem/stromal cells (MSCs) characteristics have been isolated from menstrual blood which lines the uterus and regenerates after each menstrual cycle. EnSCs can rapidly grow in vitro and have been shown to be positive for MSCs markers, including SSEA-4, OCT4, CD9, CD29, CD105, and CD73, although they do not express markers such as CD34, CD45, CD133 and HLA class II. Under specific conditions, EnSCs also undergo multipotent differentiation into various functional cells, including cardiomyocytes, respiratory epithelial cells, neurocytes, myocytes, endothelial and pancreatic cells, hepatocytes, adipocytes, and osteocytes [17-19]. A major advantage of EnSCs is the ease of collection via non-invasive methods and efficient, noncontroversial extraction that is autologous. The fact that this novel cell population can be routinely and safely isolated suggests a stem cell source from child-bearing women. Additionally, several recent investigations suggest an *in vivo* regenerative potential of EnSCs to treat many diseases, such as multiple sclerosis, a murine model of stroke, and models of cardiovascular disorders and liver injury [20-23]. Studies also show that EnSCs can modulate allogeneic proliferation of mononuclear cells in a dose-dependent manner which may be viewed as a potential therapeutic approach for allogeneic transplantation [24].

Considering that EnSCs have characteristics of MSCs, we hypothesized that human menstrual blood-derived EnSCs may also retain the ability to restore ovarian function. Therefore, in this study, we transplanted human EnSCs via the tail vein into chemotherapy-induced sterilized mice and measured restorative effects on ovarian function. Data suggest that transplantation of human EnSCs derived from human menstrual blood may improve ovarian function and hold promise for reproductive medicine in the future.

## Materials and methods

### Cell sources and culture

A human EnSC line was isolated from menstrual blood of a 40-year-old Chinese woman after written informed consent was obtained [19, 23]. Briefly, human menstrual blood was collected using a Divacup (E-vans Biotech, Hangzhou, China) during the first day of menstruation. Mononuclear cells were separated by Ficoll-Paque (1.077 g/mL, Fisher Scientific, Portsmouth, NH) density-gradient centrifugation according to the manufacturer’s instructions. The purified mononuclear cells were cultured in the Chang Medium [18] overnight at 37 °C in 5 % CO_2_. Cells were trypsinized, subcultured, and passaged every 4–6 days. Cells were used for experiments until they reached 80–90 % confluence.

Human cells project were approved by the Ethics Committee of the International Peace Maternity and Child Health Hospital, Shanghai Jiaotong University, Shanghai, China (Permit number: 2013–11).

### Flow cytometry

Expression of cell surface markers CD29, CD90, CD34, CD45, CD117, HLA-DR, and OCT4 were measured in EnSCs. Cells (1 × 10^6^) were suspended in 2% BSA/PBS and labeled with PE anti-human CD90, PE anti-human CD29, PE anti-human CD34, FITC anti-human CD45 (all purchased from Beckman Coulter Company, France), and anti-human OCT4 (eBioscience, San Diego, CA). Immunoglobulin isotype (eBioscience) incubation was performed as a negative control. Flow cytometry was performed using a FC500 flow cytometer (Beckman Coulter, Fullerton, CA) and analyzed using Beckman Coulter CXP software.

### EnSC differentiation

EnSCs were cultured and induced with human mesenchymal stem cell functional identification kit (R&D System, Minneapolis, Minnesota, USA) as instructed by the manufacturer. Cells were then fixed in 4 % formaldehyde. For osteogenic marker, cells were stained with Alizarin red for 5 min. For chondrogenic differentiation, cells were stained with Alcian blue for 30 min. For adipogenic differentiation, cells were stained with Oil Red O for 30 min [19].

### Animals

C57BL/6 wild-type female mice (n = 240; 6 weeks of age; 18.3 ± 0.1 g) were purchased from Shanghai SLAC Laboratory Animal Co., Ltd. Of these, 160 were sterilized by intraperitoneal injection of busulfan (Sigma-Aldrich, St. Louis, MO, 30 mg/kg; resuspended in DMSO) and cyclophosphamide (Sigma-Aldrich, 120 mg/kg; resuspended in DMSO) [15, 16] and were observed for 1 week (n = 160, 18.2 ± 0.3 g). Then, 80 age-matched females injected with DMSO only served as non-sterilized normal controls (n = 80, 18.2 ± 0.2 g). All animal procedures were approved by the Institutional Animal Care and Use Committee of Shanghai, and were performed in accordance with the National Research Council Guide for Care and Use of Laboratory Animals. The protocol was approved by the Committee on the Ethics of Animal Experiments of Shanghai Jiaotong University. All surgery was performed under sodium pentobarbital anesthesia, and mice were placed on a heating plate to maintain body temperature till the recovery.

### Animal methods

EnSCs were grown to a density of 85 %, and were infected with enhanced green fluorescent protein (EGFP, a gift from Tianjin Liu) [25] with lentivirus at multiplicity of infection (MOI) of 10. After growing for another two days, EnSCs were examined by flow cytometry. The overall cell transfection rate was determined to be greater than 95 %, Then EnSCs were washed three times and trypsinized, neutralized in 10 % FBS, washed with phosphate-buffered saline (PBS) and resuspended in the culture medium.

Animals were anesthetized with pentobarbital sodium (45 mg/kg, ip). Next, approximately 20 μL of cell suspension containing 2 × 10^6^ EnSCs of 5th passages (Chemoablated with EnSCs group; n = 80), or 20 μL of culture medium (Chemoablated group; n = 80) were injected into the recipients via the tail vein 1 week after chemotherapy. Non-sterilized mice were untreated controls (n = 80).

One week after injection of EnSCs, vaginal smears were obtained at 9:00 am daily for 2 months from untreated control, chemoablated with EnSCs groups, and chemoablated group animals to observe diestrus, proestrus, estrus and metestrus. Papanicolaou staining was used to evaluate the estrous cycle as previously described [26, 27] and animals in each sub-stage were quantified.

After intravenous EnSC injection, animals of three groups were weighed. Then, 1 to 8 weeks after cell transplantation, animals were euthanized under anesthesia. Organs (heart, liver, kidney, spleen, and ovary) from all animals were collected and fixed with 4 % paraformaldehyde (4 °C, overnight), dehydrated through a graded ethanol series, vitrified in xylene, embedded in paraffin, and then stained with hematoxylin and eosin (H&E).

For mating trials, 8 weeks after chemotherapy, EnSC-treated (n = 10) and chemoablated females (n = 10) were housed with proven fertile males for 3 months at a ratio of 1:2. Then, the number of offspring per litter was recorded.

### Live imaging of transplanted EnSCs in mice

Next, 6 h to 14 days after tail vein injection of GFP transfected EnSCs, treated (n = 18) and untreated mice (n = 18) were screened with a live imaging system (eXplore Optix, GE Company) to characterize, quantify and visualize GFP (+) cells. The detected signal is very weak because of the fur, so the mice were anesthetized and abdominal cavity were opened to detect the signal.

### Immunofluorescent staining

Ovaries from three groups animals were fixed with an optimal cutting temperature (OCT) compound (Sakura Finetek Middle East, Dubai, United Arab Emirates) and 5-μm-thick fresh sections were made. Slides were washed twice with PBS and incubated with blocking solution for 30 min at room temperature. Sections were incubated overnight at 4 °C with rabbit polyclonal anti-GFP (dilution 1:200; Chemicon, Billerica, MA), rabbit anti-human polyclonal FSHR (1:100, Abcam, Cambridge, UK), mouse anti-human nuclear monoclonal antibody (dilution 1:50; Millipore), mouse monoclonal anti-BrdU (1:200 dilution; Lab Vision Corporation) or rabbit anti-human polyclonal MVH (VASA homolog, MVH; DEAD box protein 4, DDX4; 1:100, ab13840, Abcam) [27]. Then, sections were washed three times with 1 × PBS, and probed with FITC-labeled IgG (1:200, Santa Cruz, CA) or Rhodamine (TRITC)-labeled IgG (1:100, Invitrogen, Carlsbad, CA). Fluorescent images were obtained with a Leica DMI3000 microscope.

### Histomorphometric analysis of the ovarian follicles and germline stem cells (GSCs)

Ovaries were fixed by 10 % formalin, paraffin embedded, serially sectioned (8 μm), aligned in order on glass microscope slides, and stained with hematoxylin and picric methyl blue. The number of non-atretic or atretic primordial, primary and preantral follicles was then determined, as described previously [12]. Immature follicles were scored as atretic if the oocyte was degenerating (convoluted, condensed) or fragmented. Grossly atretic immature follicles lacking oocyte remnants were not included in the analyses.

For GSCs counting, recipient mice were injected with BrdU (50 mg/kg) and ovaries were collected 1 h later for dual immunofluorescent analysis of BrdU incorporation and MVH expression as described above. The presence of BrdU–MVH double-positive cells in the ovarian was assayed in different groups.

### Statistical analysis

Animal weight, folicles and GSCs counting were quantified across groups and compared by ANOVA using Microsoft Excel software and differences were considered statistically significant when *P* < 0.05. Offspring distribution between groups was assayed using Kruskal-Wallis test. Differences were considered statistically significant when *P* < 0.05 or < 0.01 (pairwise comparison).

## Results

### Characterization of established human EnSCs

**Fig 1:**
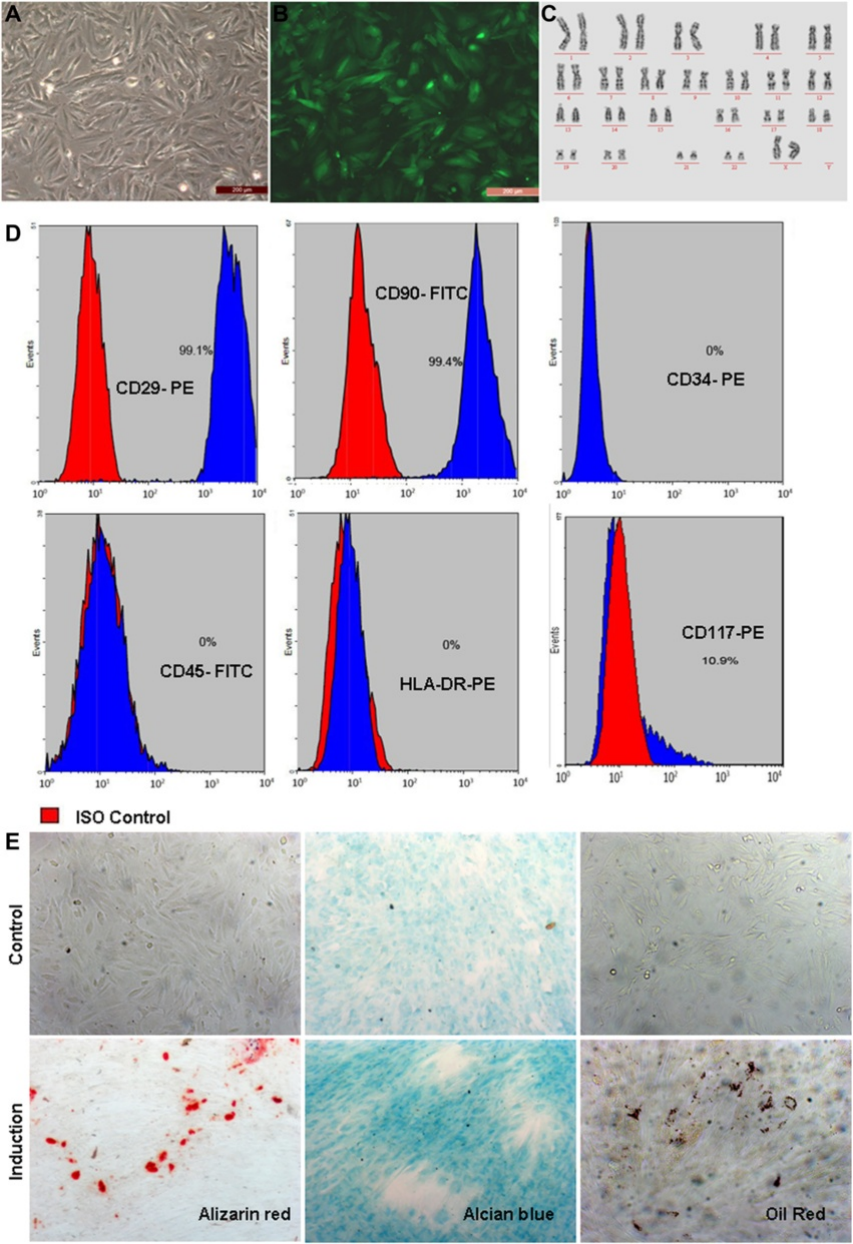
Morphology, phenotype and pluripotency of human EnSCs. **a** Cultured EnSCs appear to have stromal cell morphology. **b** GFP-transfected EnSCs. **c** Normal chromosomes expressed by cells as measured by karyotype analysis at passage 20. **d** Mesenchymal stem cell marker expression in EnSCs as measured by flow cytometry. **e** EnSCs can differentiate into adipocytes (oil red), chondroblasts (alcian blue) and osteoblasts (alizarin red) under standard *in vitro* differentiating conditions (original magnification, 100x). Scale bars = 200 μm (**a, b**)

Human EnSCs line was derived from menstrual blood of a 40-year-old Chinese woman [19, 23]. The cells grew rapidly *in vitro* and were morphologically similar to fibroblast-like cells (Fig. 1A-B) and the double time is 24 hours. Cells were diploid without chromosomal aberrations as measured by karyotype analysis at passage 20 (Fig. 1C).

Flow cytometry was used to detect the phenotype of the 5th passage of EnSCs. Fig. 1D depicts cell surface marker data. Also, human EnSCs cells were multipotent, as indicated by their ability to differentiate into adipocytes, chondroblasts and osteoblasts (Fig. 1E).

These data suggest that human EnSCs had characteristics of MSCs.

### Human EnSCs transplantation increased weight and improved cyclicity of sterilized mice

**Fig. 2:**
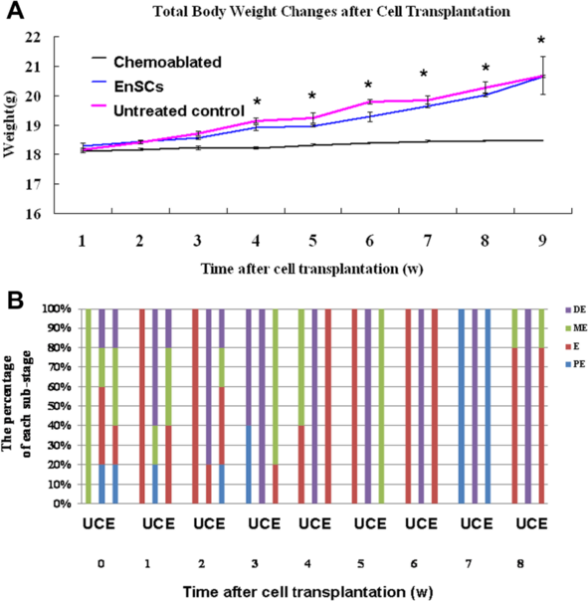
Human EnSCs transplantation increased animal weight and restored cyclicity in sterilized mice. **a** Weight of untreated mice did not increase over the study period. Mice of EnSCs-treated groups weighed significantly more compared with chemoablated mice from the 4th week onward (*P <0.01); however, there was no significant difference between untreated control and EnSCs-treated groups (*P* > 0.05). **b** The percentage of substages of diestrus (DE), proestrus (PE), estrus (E) and metestrus (ME) in different groups. Cyclicity was similar to normal animals 4 weeks after cell transplantation, whereas changes in untreated control animals were maintained at diestrus throughout the experiment. U, Untreated control; E, Chemoablated with EnSCs transplantation group, C, Chemoablated group

To assess whether human EnSCs transplantation could restore ovary function, wild-type female mice were sterilized by pre-treatment with cyclophosphamide and busulfan to destroy the existing pre- and post-meiotic germ cell pools as previously reported [15, 16]. These mice were used as recipients. EnSCs grown to 85% density were infected with green fluorescent protein (GFP) lenti-virus (Fig. 1A-B). After infection and culture for 1 week, 2 × 10^6^ cells were transplanted by tail vein injection into recipient females 7 days after chemotherapy. No transplant-related deaths occurred. We observed a significant increase in body weight in EnSCs-treated animals compared with chemoablated group from the 4th week onward after cell transplantation (*P* < 0.01, Fig. 2A). There was no difference in untreated control and EnSCs-treated group (Fig. 2A).

**Fig. 3:**
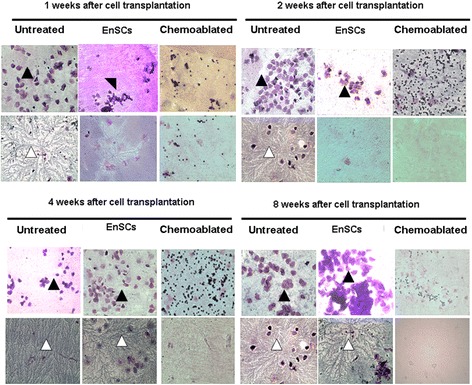
Vaginal exfoliative cell smear and cervical mucus crystallization indicating estrous cycles of mice in different groups and at different observed time points. At 2, 4, and 8 weeks after cell transplantation, the typical cornified epithelial (black arrow) and typical ferning patterns (white arrow) were observed in EnSCs-treated mice, whereas cyclicity in Chemoablated animals remained unchanged

One week after transplantation, vaginal smears from chemoablated with EnSCs group began to change and did so throughout the experiment. All diestrus, proestrus, estrus and metestrus cycle stages were observed in EnSCs-treated animals and animals resembled untreated control animals 4 weeks after cell transplantation. However, cyclicity in chemoablated animals was not as regular as normal animals. Specifically, estrus cycles were longer or shorter, and animals remained at diestrus throughout the experiment (Figs. 2B and 3).

**Fig. 4:**
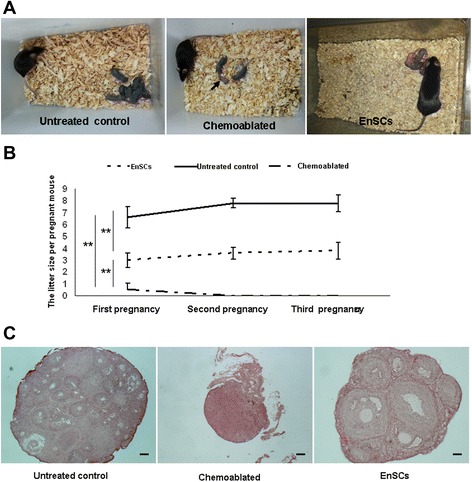
EnSCs transplantation restores fertility in mice treated with chemotherapy. Reproductive outcomes were assessed over three successive mating rounds in different mouse groups. **a** Offspring obtained by mating after EnSCs transplantation into mice sterilized by chemotherapy compared with chemoablated group and untreated control. Note the stillbirth in a chemoablated mouse (Arrow). **b** Mean litter size per pregnant mouse for three litters in each group. Data represent means ± standard error. n = 10 per group. **P < 0.001. **c** Midline histological sections of ovaries removed after the mating rounds and stained with H&E. Scale bars = 100 μm

The changes of major organs affected by B/C treatment with H&E staining were also evaluated. Besides ovaries, the lung, heart, liver and spleen were also damaged 7 days after chemotherapy. H&E staining showed that inflammation and edema in cardiac fibroblasts, glomerular congestion and atrophy, mesenchymal edema and congestion, hepatic inflammatory cell infiltration, and spleen lymphocyte proliferation. However, 14 days after chemotherapy, organ inflammation decreased to various degrees (Additional file 1: Figure S1). However, ovaries affected by B/C treatment were seriously and irreversibly damage (oocyte loss, fibrosis, and sterility) (Fig. 4C and Additional file 1: Figure S1E).

Data indicate that human EnSCs transplantation increased the weight of chemotherapy-sterilized mice and the return of an estrous cycle after cell transplantation indicated a potential ovarian functional recovery.

### Human EnSCs transplantation re-established fertility in sterilized mice

To assess the influence of human EnSCs transplantation on fertility, sterilized female mice were mated with untreated males with proven fertility. According to cyclicity results, 8 weeks after chemotherapy, mice were chosen for the mating trial and observed for 3 months. The total number of pregnancies per group and pups per pregnancy were recorded. All reproductive outcomes measured indicated that chemotherapy reduced reproductive capability, including incidence of pregnancy and reduced the average number of pups per pregnancy of each female mouse. Both EnSCs-treated animals (n = 10) and untreated control (n = 10) acquired three successful pregnancies, whereas chemoablated mice had only one pregnancy including stillbirth (Fig. 4A and Additional file 2: Table S1). Sterilized female mice that underwent EnSCs transplantation had more pups per mouse than chemoablated group (Fig. 4B and Additional file 2: Table S1). However, compared with untreated normal control, chemoablated with EnSCs group had significantly fewer numbers of pups per female mouse (Fig. 4B and Additional file 2: Table S1).

Histological examination of midline ovarian sections after mating rounds revealed that ovaries of chemotherapy-treated mice were smaller than those of chemoablated with EnSCs group or untreated control (Fig. 4C). In addition, chemoablated with EnSCs group had numerous oocytes at all stages of development, similar to ovaries of non-sterilized mice; however, no follicles (with the exception of stroma) were observed in ovaries of chemoablated mice (Fig. 4C).

Thus, these data indicate that human EnSCs transplantation restored fertility in sterilized female mice.

### Human EnSCs infiltrated into chemically-damaged murine ovarian tissue and differentiated into granulosa cells

**Fig. 5:**
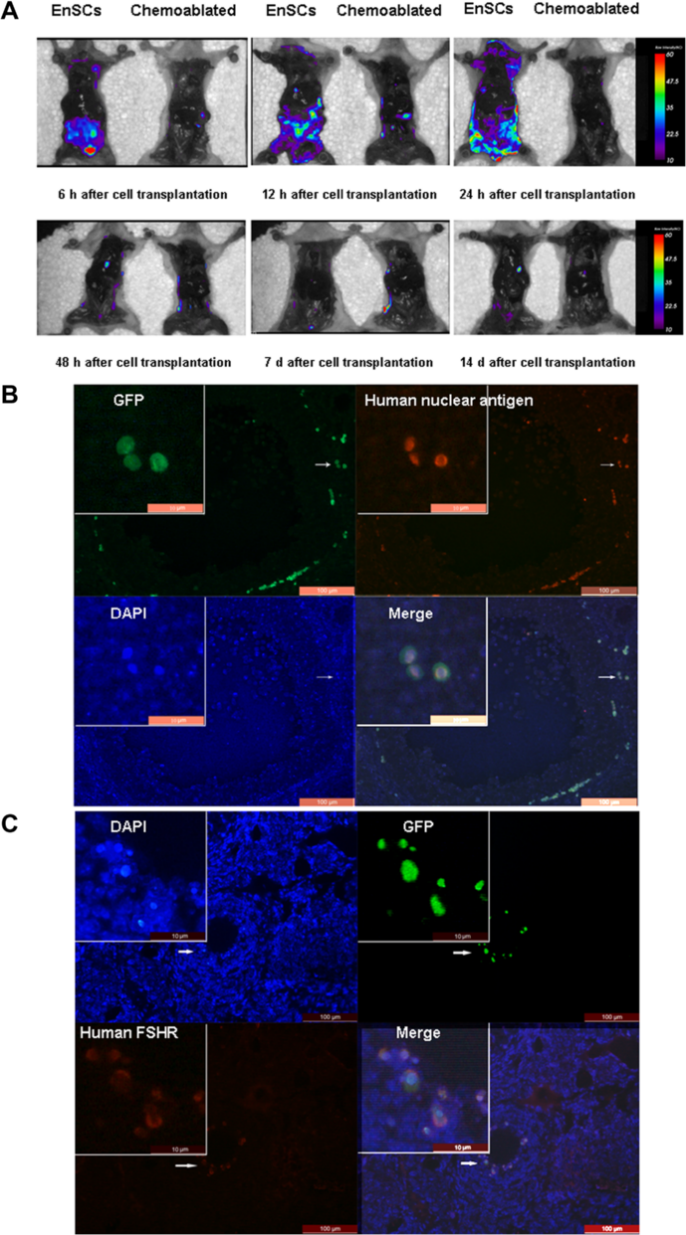
Grafted human EnSCs were detected *in vivo*. **a** Sterilized mice after tail vein cell transplantation were screened by live imaging (eXplore Optix, GE Company) for the identification of GFP-positive cell tracking *in vivo*. GFP (+) cells first entered the pelvic organs 6 to 12 h after transplantation, then migrated to chest organs 24 h after transplantation. However, the signal was too weak to be detected 48 h after cell transplantation and few signals were collected in pelvic organs 7 days after cell transplantation. Chemoablated mouse as negative control. **b** Human nuclear antigen was expressed in antral follicles in recipient ovaries and co-localized with GFP staining 2 months after EnSCs transplantation. **c** GFP staining was co-localized with human FSHR staining in antral follicles of recipient ovaries 2 months after EnSCs transplantation. Scale bars: (**b, c**) 100 μm; (**b, c** insets) 10 μm

As we previously reported [15, 16], human amniotic fluid stem cells or amniotic epithelial cells transplantation has the potential to improve ovarian function of sterilized mice. We hypothesized that human EnSCs could home and integrate into damaged ovarian tissue. To elucidate how cells homed *in vivo* after tail vein cell transplantation, sterilized mice were screened by live imaging to identify GFP (+) cell tracking 6 h to 14 days after cell transplantation. The GFP (+) cells first entered the pelvic organs 6 to 12 h after transplantation, then migrated to the chest 24 h after transplantation; however, the signal is too weak to be detected 48 h after cell transplantation and few signals were detected in pelvic organs 7 days after cell transplantation. Background signals were detected in chemoablated mice (Fig. 5A).

Immunofluorescent studies of recipient ovarian tissues were assayed to detect human cell tracking. Although GFP signal could not be detected 7 days after transplantation by live imaging, GFP-stained cells were found in ovarian tissue stroma 2 months post-transplantation. To confirm that GFP (+) cells in recipient ovaries was derived from grafted human EnSCs, we performed double-staining with GFP and human specific nuclear antigen in recipient ovarian sections 2 months after EnSCs transplantation. GFP (+) staining co-localized with human anti–nuclear staining in ovarian stroma (Fig. 5B).

To characterize grafted cells, human follicle-stimulating hormone receptor (FSHR) was used as both a granulosa cell marker and a human cell tracking marker. Data show that double staining of human FSHR and GFP was detected in cells proximal to ovarian tissue eggs 2 months after EnSCs transplantation (Fig. 5C and Additional file 3: Figure S2 for negative control). Furthermore, an immunochemical assay revealed that human FSHR staining patterns were found in cells proximal to eggs within follicles 2 months after EnSCs transplantation, a finding consistent with immunostaining patterns (Additional file 4: Figure S3).

The frequency of GFP+/human nuclear antigen (HNU) cells and GFP+/human FSHR+ cells in the EnSCs transplantation group per number of sections are depicted in Additional file 2: Table S2. These results strongly suggest that a portion of EnSCs-derived cells grafted to chemotherapeutically murine ovarian tissue and may have differentiated to granulosa cells.

### Human EnSCs administration improved renewal of germline stem cells in sterilized mice

Recently GSCs have successfully been isolated from ovaries of neonatal and adult mice as well as from human ovaries, which challenges the viewpoint that the bank of ovarian oocytes is not renewed in postnatal female mammals [27-29]. However, there is still lack of knowledge of genesis and development of GSCs in adult ovary. To identify and confirm whether EnSCs affect oogenesis in sterilized mice, GSCs were immunohistochemically quantified in mouse ovaries from different treatment groups. Morphological and histological analysis of 5’-bromodeoxyuridine (BrdU) and mouse vasa homologue (MVH) protein double-positive cells were used to identify GSCs [27-29].

First, we located ovarian cells positive for MVH protein, which is expressed exclusively in germ cells, using immunohistochemistry. Similar to previous reports [30], MVH cytoplasmic stained cells were observed near the surface of ovaries (Additional file 5: Figure S4). To assess the proliferative potential of MVH-positive ovarian cells, female mice were injected with BrdU, and ovaries were collected 1 h later for dual immunofluorescence analysis of BrdU incorporation and MVH expression.

Mice were sterilized with chemotherapy, observed for 1 week, and then EnSCs were transplanted. One week later (observation period), mice were 8 weeks of age and at this time, mice were observed for an additional 8 weeks. To better define germ-cell dynamics in EnSCs-treated female mice, nonatretic and atretic follicles were quantified in ovaries of C57BL/6 mice (untreated control, chemoablated group and chemoablated with EnSCs group). Analysis of non-atretic quiescent (primordial) and growing (primary, preantral, and antral) follicle numbers revealed that immature follicles were gradually lost after chemotherapy. However, EnSCs transplantation prevented the loss of various follicular stages and diminished the number of atretic follicles during this 8-week period (Additional file 6: Figure S5).

**Fig. 6:**
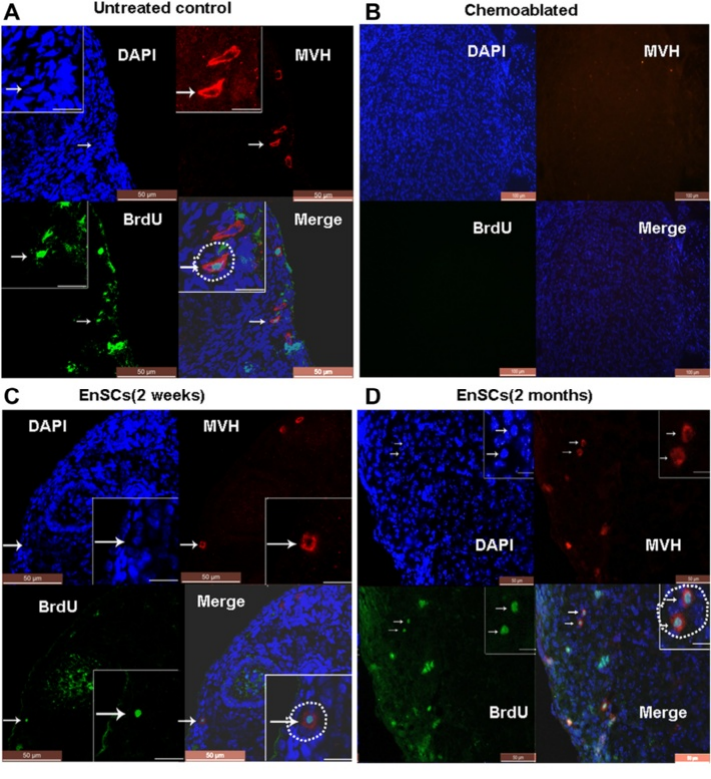
EnSCs transplantation improves GSCs proliferation in sterilized ovaries. Dual immunostaining for BrdU (green) and MVH (red) of GSCs (arrow) were observed near the surface epithelium of mouse ovaries in: (**a**) Untreated control (**b**) Chemoablated group (negative control) (**c**) EnSCs transplantation for 2 weeks (**d**) EnSCs transplantation for 2 months. A, C, D: Scale bars = 50 μm; insets = 10 μm; B: Scale bars = 100 μm

**Fig. 7:**
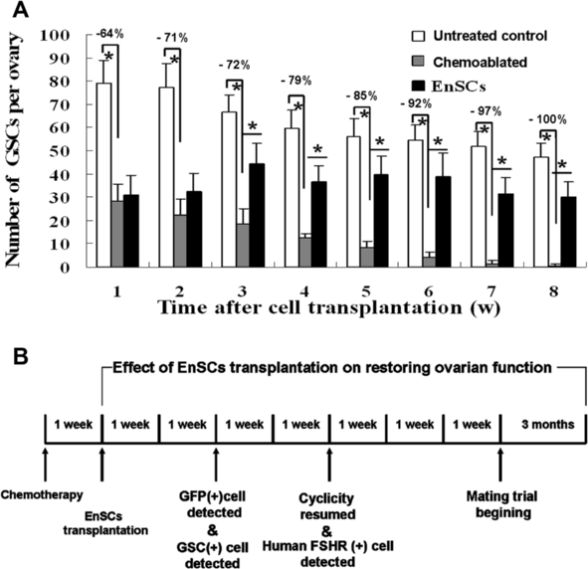
**a** Comparison of GSCs in ovaries of untreated control, chemoablated group or EnSCs-treated mice (mean ± standard error, n = 4–5 mice per data point, *P <0.01). **b** A schematics showed timing of chemotherapy, transplantation, cyclicity resumed, GFP (+) cells, GSCs (+) cells and FSHR (+) cells detected

The presence of BrdU–MVH double-positive cells near the ovarian surface epithelium was observed (Fig. 6) and the numbers of GSCs in different groups were assayed. In normal mice ~78 GSCs per ovary were found in 8-week-old female mice and this number gradually declined to ~56 when the mice were 15 weeks of age, revealing that the pool of GSCs degenerating under normal conditions. However, in ovaries of sterilized females GSCs pools decreased 64 % one week after chemotherapy with complete GSCs loss occurring 8 weeks after chemotherapy. In treated mice, after EnSCs transplantation, GSCs per ovary increased and plateaued. Ovaries of treated mice had 78.3 % of the GSCs pool present in normal controls 8 weeks after EnSCs transplantation (Fig. 7A).

Thus, EnSCs transplantation reduced the depletion of GSCs caused by chemotherapy.

## Discussion

Extraction of EnSCs from menstrual blood was first reported in 2008 [18] and EnSCs have also been examined in diverse preclinical small animal models of ischemic stroke, hind limb ischemia, and myocardial infarction [20, 31-33]. Considering future human clinical applications, EnSCs collection is easy, non-invasive, and autologous. Recently, preliminary results of the first clinical trial of EnSCs were reported [34].

Building on this detailed work, we studied the effects of EnSCs transplantation into sterilized female mice and evaluated ovarian function. EnSCs transplantation dramatically improved body weight and cyclicity in recipient mice compared with chemoablated mice (Figs. 2 and 3). In addition, the number of litters obtained by natural mating was significantly increased with chemoablated with EnSCs group compared with chemoablated mice; although fertility recovery was not complete compared to normal untreated controls (Fig. 4). Recently similar results were reported that human endometrial mesenchymal stem cells could restore ovarian function by improving the host ovarian niche [35], however, less was paid attention to the physiological and pathological effects in the sterilized mice following cell therapy.

Chemotherapeutic drugs such as busulfan and cyclophosphamide can cause prolonged and often irreversible azoospermia and ovarian damage in mice and humans. Busulfan and cyclophosphamide (B/C)-treated mice had greater apoptosis and germ cell depletion in the ovary [36]. However, few studies have focused on the physical conditions whereby chemotherapy induces these effects. We observed that animal treatment with B/C resulted in weight loss and irregular cyclicity in female mice (Figs. 2 and 3). We first noted that major organs were affected by B/C treatment. Inflammation and edema were observed in cardiac fibroblasts, liver, kidney and spleen 7 days after chemotherapy; however, 14 days after chemotherapy, organ inflammation decreased in various degrees (Additional file 1: Figure S1). In contrast, ovaries affected by B/C treatment had serious, irreversible and persisted damage: producing oocyte loss, fibrosis, and sterility (Fig. 4C and Additional file 1: Figure S1). These data show that B/C treatment chiefly affected ovarian function in female mice.

Many animal and human studies indicate a beneficial effect of MSCs infusion or implantation for tissue and organ repair. Evidence suggests that MSCs locate to sites of tissue damage when infused intravenously, after which they either engraft to damaged tissue or secrete bioactive molecules that promote tissue repair [37]. For example, intravenous injection of bone marrow MSCs in a mouse model of coronary artery disease (coronary vessel ligation) significantly improved myocardial parameters after 3 weeks; however, grafted cells were not detected after 48 h [38]. In this study, we analyzed the distribution of transplanted EnSCs, and we observed that cells localized to pelvic organs first after intravenous tail vein injection in sterilized mice. Also cells could not be detected after 48 h. However, 2 months after transplantation, GFP-stained cells were detected in recipient mice and some of these cells may have transdifferentiated into granulosa cells as indicated by human FSHR and GFP double-staining. A previous study suggests that cyclophosphamide (CTX) metabolites may induce apoptosis of granulosa cells and induce ovarian toxicity [4]. Thus, inflammatory signals induced by chemotherapy may “home” EnSCs to sites of ovarian injury and transdifferentiate into granulosa cells. We have published similar results using either human amniotic fluid cells transplanted by cell injections into damaged ovaries or using human amniotic epithelial cells transplanted by the tail vein to restore ovarian function. Both treatments restored ovarian function and grafted cells were detected in recipient ovaries [15, 16]. However, more evidence is needed to explain how EnSCs differentiate into granulosa cells.

The importance of proliferative germ cells for replenishing postnatal follicle pools has been verified with busulfan, a germ-cell toxicant widely used in spermatogonial stem-cell characterization in male mice. Busulfan could specifically target GSCs and spermatogonia and lead to spermatogenic failure in the testis [39-41]. Further studies showed that a single injection of busulfan (30 mg/kg, ip) plus cyclophosphamide (120 mg/kg) (B/C) was sufficient to sterilize female mice and that the resultant germ cell depletion was irreversible [36]. Recently, the identification and isolation of female GSCs from mouse and human ovaries supports the concept that mammalian ovaries generate new oocytes and follicles during the reproductive period [27, 29]. To elucidate the biology of female GSCs induced by B/C treatment, we firstly measured the distribution of GSCs and documented follicular stages in different animal treatment groups. Consistent with previous reports [28], we observed that GSCs were near the ovarian surface. Histomorphometric studies in 8-week-old female mice revealed the presence of 78 ± 8 such cells per ovary and these cells decreased as mice aged (Fig. 7).

B/C treatment destroys the GSCs pool, GSCs pools in ovaries of sterilized females decreased 64 % one week after chemotherapy with complete GSCs loss occurring 8 weeks after chemotherapy (Fig. 7). However, if EnSCs were transplanted a week after chemoablation, GSCs depletion was attenuated. This suggests that EnSCs transplantation restores GSCs pools. Folliculogenesis requires a carefully orchestrated cross talk between germ cells and the surrounding somatic cells, however, chemotherapy could destruct the ovarian niches, decrease granulosa cell function and induce ovarian toxicity [1-4]. Herein, we observed that some transplanted stem cells differentiated into granulosa cells which existed in the location of cumulus cells (Fig. 5 and Additional file 4: Figure S3). Cumulus cells are a specialized type of granulosa cell, distinct from the mural granulosa cells that line the antrum in developing follicles. Cumulus cells were considered to play a critical role in the reproductive potential of oocytes and embryos [42]. Although fewer than 20 % GFP+/human FSHR+ cells were detected in the EnSCs transplantation group (Additional file 2: Table S2), improvements such as restoration of cumulus cells may improve the ovarian niche, stimulate renewal of GSCs or activate primodial follicles then lead to follicle development. Also, EnSCs, as MSCs, may have a role in improving damaged immune systems via a paracrine mechanism [43]. Further research is warranted to investigate the mechanisms.

Follicle counts conducted after B/C treatment reveal vastly reduced primordial, primary and secondary follicles, whereas EnSCs transplantation recovered the reserve of primordial and growing follicles (Additional file 6: Figure S5). No evidence exists to suggest that EnSCs can differentiate into GSCs or oocytes in recipient mice. Herein the GSCs pool and primordial and growing follicles after EnSCs transplantation did not approach those numbers in normal female mice of the same age. This observation may explain why EnSCs transplantation did not completely restore fertility in chemotherapy-induced sterilized mice.

Menstrual blood-derived MSCs are attractive because they are easy to collect (compared to bone marrow aspiration), have longevity in culture, and can be subcultured to 10–20 passages with retention of a normal karyotype. We observed that these cells retained multipotency as evidenced by their ability to differentiate into adipocytes, chondroblasts, and osteoblasts (Fig. 1). These cells are currently being assessed in diverse applications as an allogeneic immunologically naive cell source [32, 33]. Finally, these cells can be harvested from women during their child-bearing years, offering up to 12 opportunities a year for collection. This would offer researchers ample time to collect and store cells for multiple reproductive therapies, including POF induced by chemotherapy.

## Conclusions

EnSCs derived from human menstrual blood have characteristics of MSCs which is able to undergo multipotent differentiation under specific conditions. As an autologous cell source of stem cells, EnSCs transplantation may be a suitable clinical strategy for preserving ovarian function or fertility in POF/POI patients induced by chemotherapy.

## Additional files

Additional file 1: Figure S1. Histological evaluations of major organs in untreated control and normal control animals by HE staining. A1) Normal cardiac fibroblasts. A2) Edematous cardiac fibroblasts 7 days after chemotherapy. A3) Edematous cardiac fibroblasts 14 days after chemotherapy. B1) Normal glomerular and tubular structure. B2) Congestion of tubular structure with glomerular atrophy 7 days after chemotherapy. B3) Kidney restoration 14 days after chemotherapy. C1) Normal histological liver division into lobules (the center of the lobule is the central vein) (V). C2) Edema and congestion in mesenchymal and inflammatory cell infiltration in liver C3) Recovered liver tissue 14 days after chemotherapy. D1) The spleen is a large lymphoid organ comprised of “white pulp” (arrow) and “red pulp” (arrowhead) respectively. D2) Spleen lymphocytes proliferated after chemotherapy. D3) Restoration of spleen tissue 14 days after chemotherapy. E1) Normal ovarian tissue. E2) Atretic follicles (arrow) and fibrosis in the damaged ovary 7 days after chemotherapy. E3) Extensive fibrosis in the ovarian stroma 14 days after chemotherapy. Magnification, 100×.

Additional file 2 **Table S1.** Summary of female fertility study. **Table S2.** Quantification of GFP+ cells detected in different groups.

Additional file 3: Figure S2 Negative control image for GFP, Human nuclear antigen, and human FSHR. A) Human nuclear antigen and GFP were not detected in untreated controls. B) Human FSHR and GFP were not detected in normal controls. Scale bars = 100 μm.

Additional file 4: Figure S3. Grafted cells detected by immunochemistry against human FSHR antigens 2 months after EnSCs transplantation. (A) Human FSHR was not detected in untreated control ovaries without EnSCs transplantation. (B) Human FSHR was not detected in normal control ovaries. (C, D) Human FSHR were detected in recipient ovaries 2 months after EnSCs transplantation. Arrows indicated positive staining. Original magnification, 100× (A, C), 200× (B, D).

Additional file 5: Figure S4. MVH stained cells were observed near the surface of mouse ovaries (Arrow). Scale bars = 50 μm; insets = 10 μm.

Additional file 6: Figure S5. Follicle counts of primordial, primary, secondary, and atretic follicles in ovaries of each group, including untreated control, Chemoablated group and EnSCs-treated animals.

## Abbreviations

EnSCs: Human endometrial mesenchymal stem cells
POF/POI: Premature ovarian failure/insufficiency
GFP: Green fluorescent protein
B/C: Busulfan and cyclophosphamide
FSHR: Human follicle-stimulating hormone receptor
BrdU: 5’-bromodeoxyuridine
MVH: Mouse vasa homologue
GSCs: Germline stem cells
