# Supplementary figures and images for: Human endometrial mesenchymal stem cells restore ovarian function through improving the renewal of germline stem cells in a mouse model of premature ovarian failure
Source: J Transl Med. 2015 May 12;13:155. doi: 10.1186/s12967-015-0516-y (PMC4490699; doi:10.1186/s12967-015-0516-y)

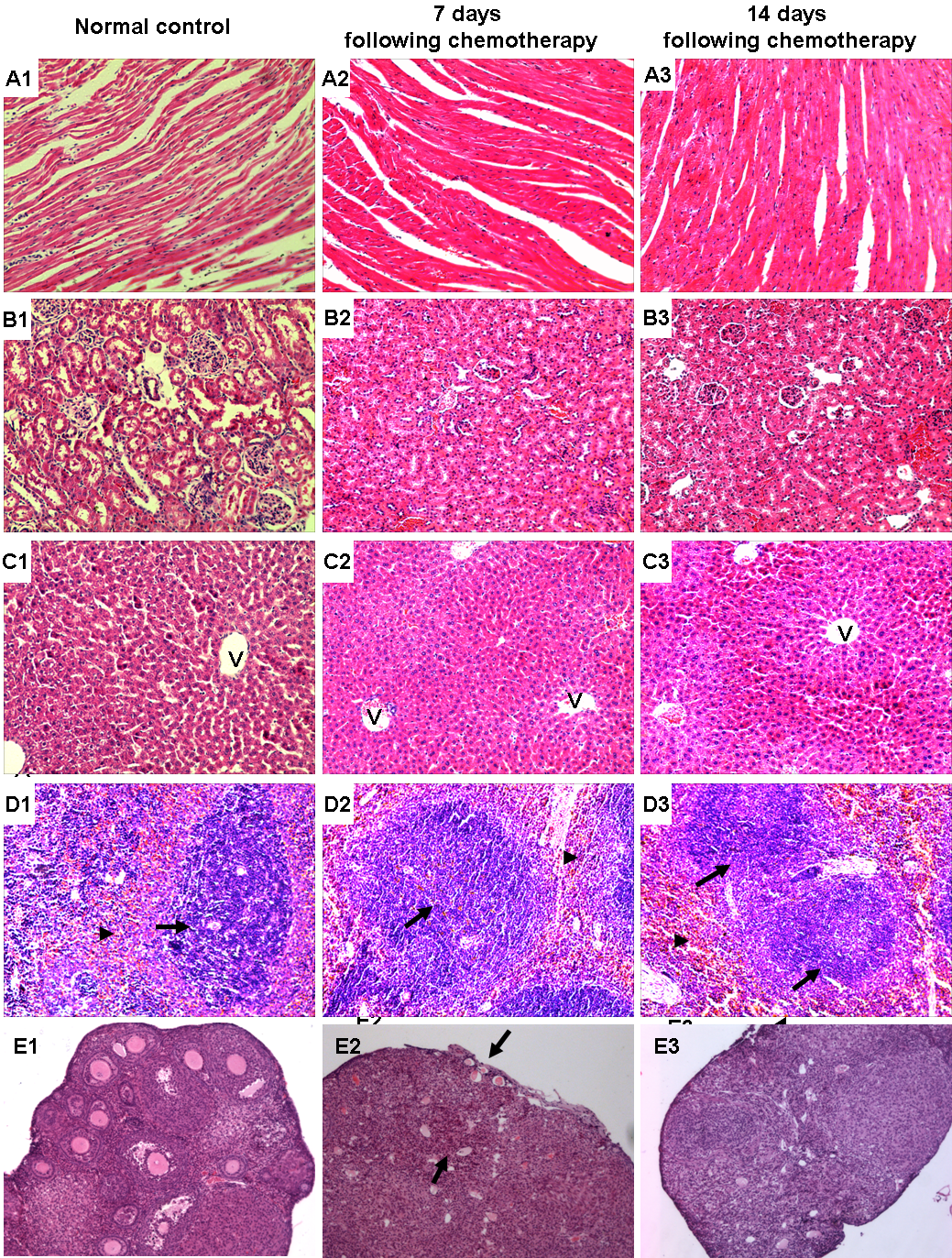

Supplement: Supplementary file 1 — Histological evaluations of major organs in untreated control and normal control animals by HE staining. A1) Normal cardiac fibroblasts. A2) Edematous cardiac fibroblasts 7 days after chemotherapy. A3) Edematous cardiac fibroblasts 14 days after chemotherapy. B1) Normal glomerular and tubular structure. B2) Congestion of tubular structure with glomerular atrophy 7 days after chemotherapy. B3) Kidney restoration 14 days after chemotherapy. C1) Normal histological liver division into lobules (the center of the lobule is the central vein) (V). C2) Edema and congestion in mesenchymal and inflammatory cell infiltration in liver C3) Recovered liver tissue 14 days after chemotherapy. D1) The spleen is a large lymphoid organ comprised of “white pulp” (arrow) and “red pulp” (arrowhead) respectively. D2) Spleen lymphocytes proliferated after chemotherapy. D3) Restoration of spleen tissue 14 days after chemotherapy. E1) Normal ovarian tissue. E2) Atretic follicles (arrow) and fibrosis in the damaged ovary 7 days after chemotherapy. E3) Extensive fibrosis in the ovarian stroma 14 days after chemotherapy. Magnification, 100×. [file 12967_2015_516_MOESM1_ESM.tiff]

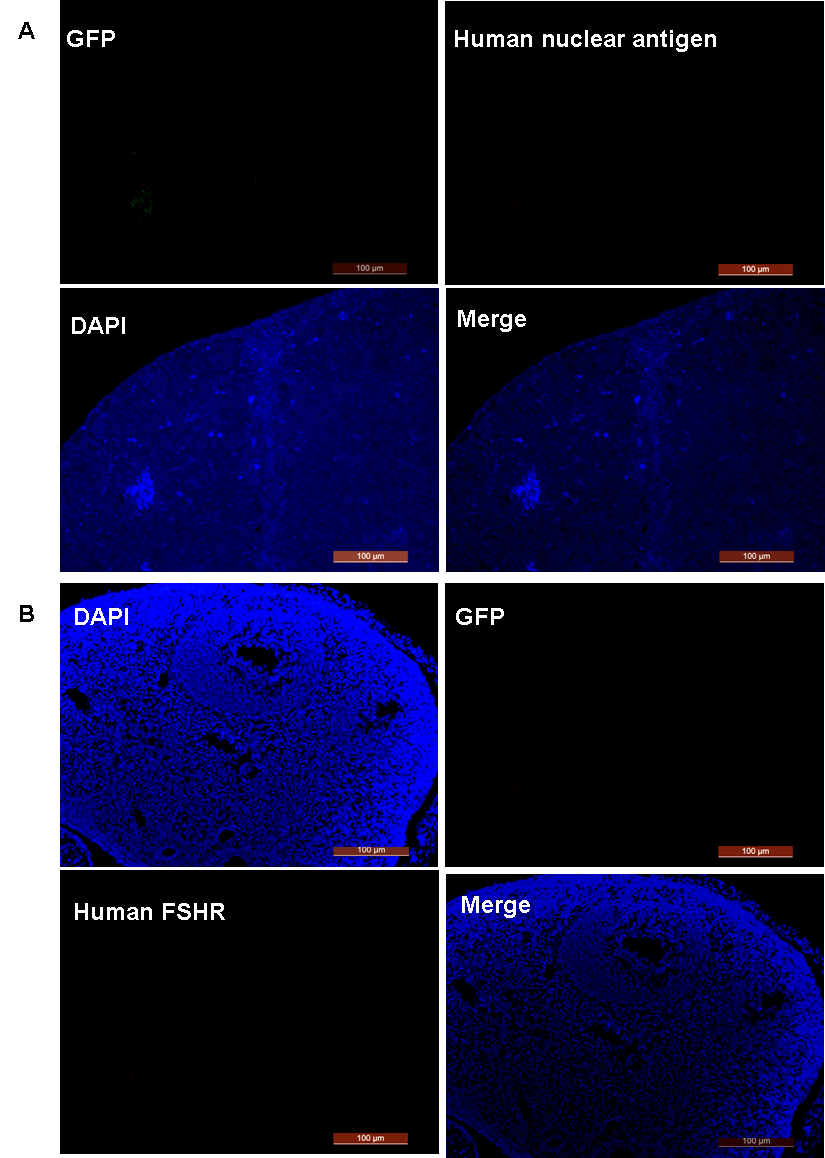

Supplement: Supplementary file 3 — Negative control image for GFP, Human nuclear antigen, and human FSHR. A) Human nuclear antigen and GFP were not detected in untreated controls. B) Human FSHR and GFP were not detected in normal controls. Scale bars = 100 μm. [file 12967_2015_516_MOESM3_ESM.tiff]

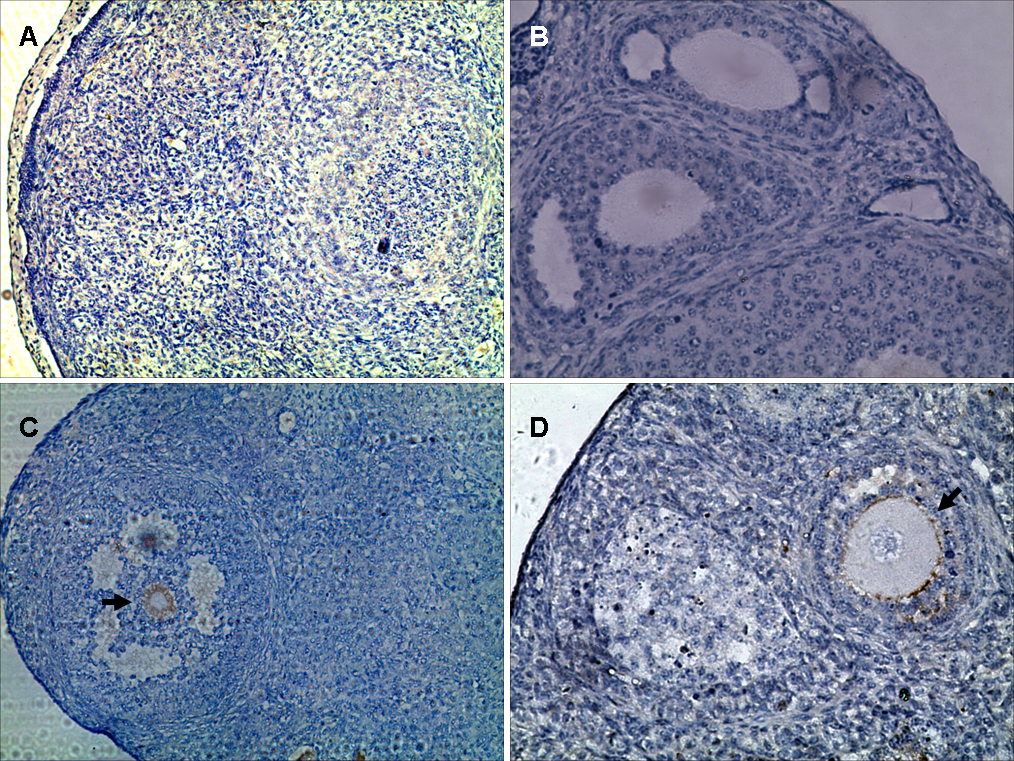

Supplement: Supplementary file 4 — Grafted cells detected by immunochemistry against human FSHR antigens 2 months after EnSCs transplantation. (A) Human FSHR was not detected in untreated control ovaries without EnSCs transplantation. (B) Human FSHR was not detected in normal control ovaries. (C, D) Human FSHR were detected in recipient ovaries 2 months after EnSCs transplantation. Arrows indicated positive staining. Original magnification, 100× (A, C), 200× (B, D). [file 12967_2015_516_MOESM4_ESM.tiff]

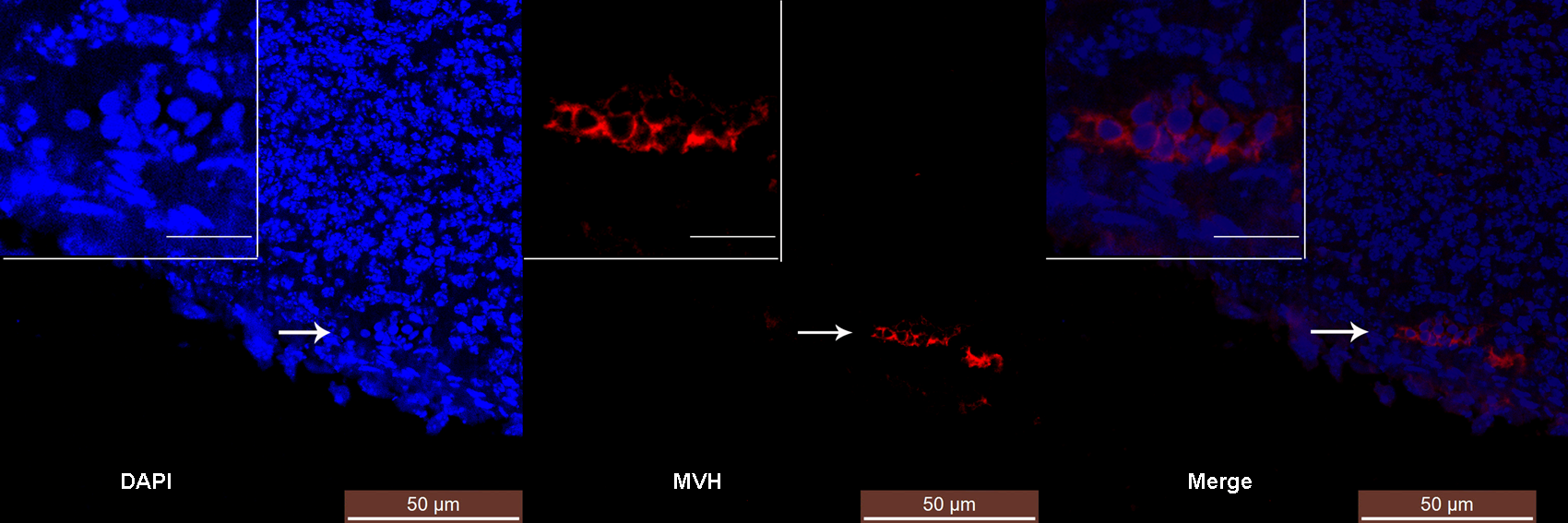

Supplement: Supplementary file 5 — MVH stained cells were observed near the surface of mouse ovaries (Arrow). Scale bars = 50 μm; insets = 10 μm. [file 12967_2015_516_MOESM5_ESM.tiff]

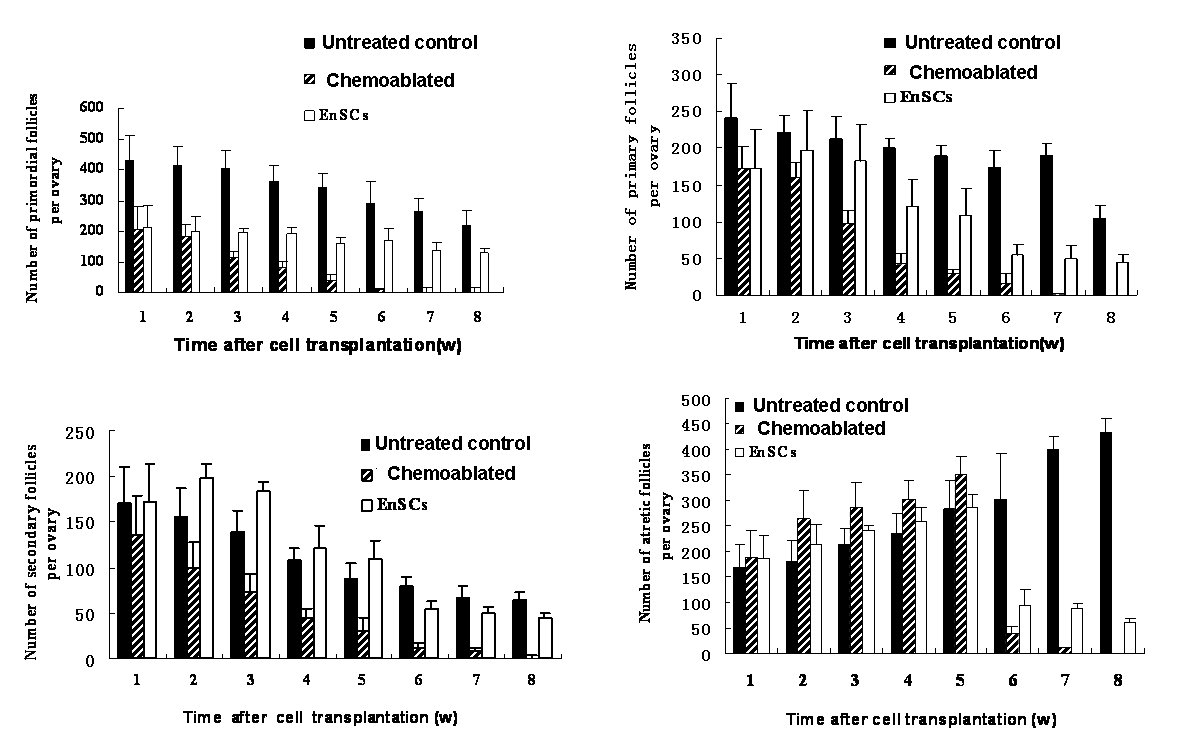

Supplement: Supplementary file 6 — Follicle counts of primordial, primary, secondary, and atretic follicles in ovaries of each group, including untreated control, Chemoablated group and EnSCs-treated animals. [file 12967_2015_516_MOESM6_ESM.tiff]
