# Supplementary material for: Human endometrial mesenchymal stem cells restore ovarian function through improving the renewal of germline stem cells in a mouse model of premature ovarian failure
Source: J Transl Med. 2015 May 12;13:155. doi: 10.1186/s12967-015-0516-y (PMC4490699; doi:10.1186/s12967-015-0516-y)
Supplement: Supplementary file 2 — Table S1. Summary of female fertility study. Table S2. Quantification of GFP+ cells detected in different groups. [file 12967_2015_516_MOESM2_ESM.doc]

| Additional file 2: Table S1. Summary of female fertility study | | | | |
| --- | --- | --- | --- | --- |
| Number | Untreated control | chemoablated with EnSCs group | chemoablated group | |
| Animals (female) | 10 | 10 | 10 |  |
| Evaluated | 10 | 10 | 10 |  |
| Died/sacrificed moribund | 0 | 0 | 0 |  |
| Animals mated | 10 | 10 | 10 |  |
| Pregnant Animals | 10 | 10 | 4 |  |
| Pregnancy Number (%) |  |  |  |  |
| Up to 3 pregnancies | 100 (10/10) | 100 (10/10) | 0** |  |
| Up to 2 pregnancies | 100 (10/10) | 100 (10/10) | 0** |  |
| Up to 1 pregnancy | 100 (10/10) | 100 (10/10) | 40 (4/10)** |  |
| Number of embryos |  |  |  |  |
| Live embryos | 208 | 109 | 3 |  |
| Mean embryos | 7.40** | 3.47** | 0.3** |  |
| Dead embryos | 0 | 0 | 6 |  |
| Mean dead embryos | 0 | 0 | 0.6** |  |

*: P < 0.01, **: P < 0.001

All of the mice in both untreated control and chemoablated with EnSCs group were pregnant, whereas only 4/10 was pregnant in chemoablated group. During the three months mating trial, all of the mice in both untreated control and chemoablated with EnSCs group acquired three successful pregnancies, whereas 4/10 in chemoablated mice had only one pregnancy including stillbirth. The mean live embryos in untreated normal control was 7.4, which was more than that in chemoablated with EnSCs group (3.47, P<0.001). The mean live embryos in chemoablated group was 0.3, which was the least compared with the other two groups (P<0.001).

Additional file 2: Table S2. Quantification of GFP+ cells detected in different groups

|  | The number of sections | The frequency of GFP+/HNU+/sections (%) | The frequency of GFP+/ human FSHR+/sections (%) |
| --- | --- | --- | --- |
| Untreated control (n = 70) | 500 | 0 | 0 |
| chemoablated group  (n = 70) | 220 | 0 | 0 |
| chemoablated with EnSCs group (n = 70) | 500 | 120/500(24.0%) | 58/500(11.6%) |

Human nuclear antigen, HNU
